# Polycyclic aromatic hydrocarbons (PAHs) cycling and fates in Galveston Bay, Texas, USA

**DOI:** 10.1371/journal.pone.0243734

**Published:** 2020-12-28

**Authors:** Gilbert T. Rowe, Harshica Fernando, Cornelis Elferink, G. A. Shakeel Ansari, John Sullivan, Thomas Heathman, Antonietta Quigg, Sharon Petronella Croisant, Terry L. Wade, Peter H. Santschi

**Affiliations:** 1 Department of Marine Biology, Texas A&M University at Galveston, Galveston, Texas, United States of America; 2 Department of Chemistry, Prairie View A&M University, Prairie View, Texas, United States of America; 3 Department of Pathology, University of Texas Medical Branch, Galveston, Texas, United States of America; 4 Department of Oceanography, Texas A&M University, College Station, Texas, United States of America; 5 Department of Marine and Coastal Environmental Science, Texas A&M University at Galveston, Galveston, Texas, United States of America; National Sun Yat-sen University, TAIWAN

## Abstract

The cycling and fate of polycyclic aromatic hydrocarbons (PAHs) is not well understood in estuarine systems. It is critical now more than ever given the increased ecosystem pressures on these critical coastal habitats. A budget of PAHs and cycling has been created for Galveston Bay (Texas) in the northwestern Gulf of Mexico, an estuary surrounded by 30–50% of the US capacity of oil refineries and chemical industry. We estimate that approximately 3 to 4 mt per year of pyrogenic PAHs are introduced to Galveston Bay via gaseous exchange from the atmosphere (ca. 2 mt/year) in addition to numerous spills of petrogenic PAHs from oil and gas operations (ca. 1.0 to 1.9 mt/year). PAHs are cycled through and stored in the biota, and ca. 20 to 30% of the total (0.8 to 1.5 mt per year) are estimated to be buried in the sediments. Oysters concentrate PAHs to levels above their surroundings (water and sediments) and contain substantially greater concentrations than other fish catch (shrimp, blue crabs and fin fish). Smaller organisms (infaunal invertebrates, phytoplankton and zooplankton) might also retain a significant fraction of the total, but direct evidence for this is lacking. The amount of PAHs delivered to humans in seafood, based on reported landings, is trivially small compared to the total inputs, sediment accumulation and other possible fates (metabolic remineralization, export in tides, etc.), which remain poorly known. The generally higher concentrations in biota from Galveston Bay compared to other coastal habitats can be attributed to both intermittent spills of gas and oil and the bay's close proximity to high production of pyrogenic PAHs within the urban industrial complex of the city of Houston as well as periodic flood events that transport PAHs from land surfaces to the Bay.

## Introduction

The environmental, human health and economic consequences of oil spills have been recently reviewed [1–4]. Highly developed and populated estuaries are some of the most vulnerable regions on the planet–both from land-sourced run-off of oil compounds [2, 5, 6] as well as from a host of petroleum transportation vessels that release oil during routine processes (e.g. tank cleaning, transfer of contents, engine maintenance) and, more noticeably, during accidents [2, 5, 7]. Oil is in fact, predicted to be one of the most detrimental sources of anthropogenic pollution to estuaries [5]. Polycyclic aromatic hydrocarbons (PAHs) are a large group of organic compounds with two or more fused aromatic rings. Low-molecular weight PAHs (two and three rings) occur in the atmosphere predominantly in the vapor phase, whereas multi-ringed PAHs (five rings or more) are largely bound to particles. Intermediate-molecular-weight PAHs (four rings) are partitioned between the vapor and particulate phases, depending on the atmospheric temperature. Particle-bound PAHs are considered to be very hazardous to human health. Benzo[a]pyrene (B[a]P) is often used as a marker for total exposure to carcinogenic PAHs, as the contribution of B[a]P to the total carcinogenic potential is high, in the range 51–64% [8].

We use the term PAHs here to mean total PAHs, in contrast to individual PAH compounds. Both low-molecular weight compounds such as benzene, naphthalene or anthracene, and high-molecular weight, PAHs compounds such as pyrene and fluoranthene, are utilized by microbes in the environment, including fungi, bacteria, cyanobacteria and phytoplankton [7, 9, 10]. Some of these in fact use PAHs as carbon source for growth and production; and/or these materials are biotransferred and/or biomagnified through food webs (see e.g., [11]).

According to [8], the human exposure of PAHs will be from both inhalation of contaminated air and consumption of contaminated food and water. Especially high exposure will occur through the smoking of cigarettes and the ingestion of certain foods (e.g. smoked and charcoal-broiled meats and fish). Food ingestion is likely to be a larger route of exposure compared to inhalation for a large section of the general population exposed to PAHs. Drinking-water and soil are generally minor sources of these compounds in the daily intake dose.

Choi et al. [8] furthermore reports that for the average American diet, the dietary intake of carcinogenic PAHs was estimated to be 1–5 μg/day, with unprocessed grains and cooked meats the greatest sources of the compounds. In an earlier American study, diet was reported to make a substantial contribution (generally more than 70% in non-smokers) to PAH intake other than occupational PAH exposure. For a non-smoking reference male (70 kg body weight), a mean carcinogenic PAH intake of 3.12 μg/day was estimated, of which dietary intake contributed 96.0%, air 1.6%, water 0.2% and soil 0.4%. In the early 1990s, the potential dose of carcinogenic PAHs for American adult non-smoking males was estimated to be 3 μg/day up to a maximum of 15 μg/day. Smokers of unfiltered cigarettes might have had a potential dose twice that of non-smokers.

**Fig 1:**
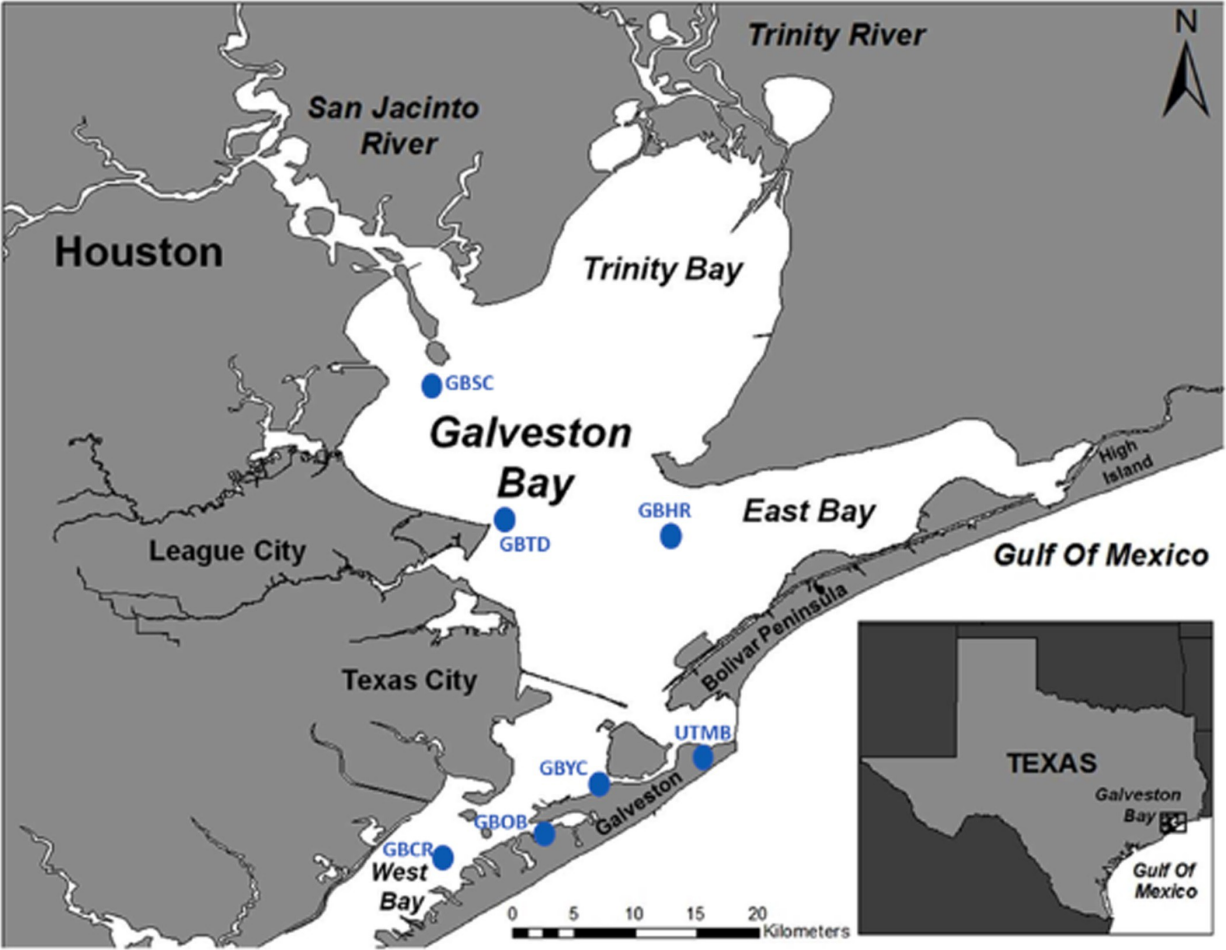
Galveston Bay located south of Houston (Texas) is the second largest estuary in the Gulf of Mexico. Oyster samples were collected from six sites in Galveston Bay including the Ship Channel (GBSC), Hanna Reef (GBHR), Yacht Club (GBYC), Todd’s Dump (GBTD), Offatts Bayou (GBOB), and Confederate Reef (GBCR) (Qian et al., (2001) [103].

**Fig 2:**
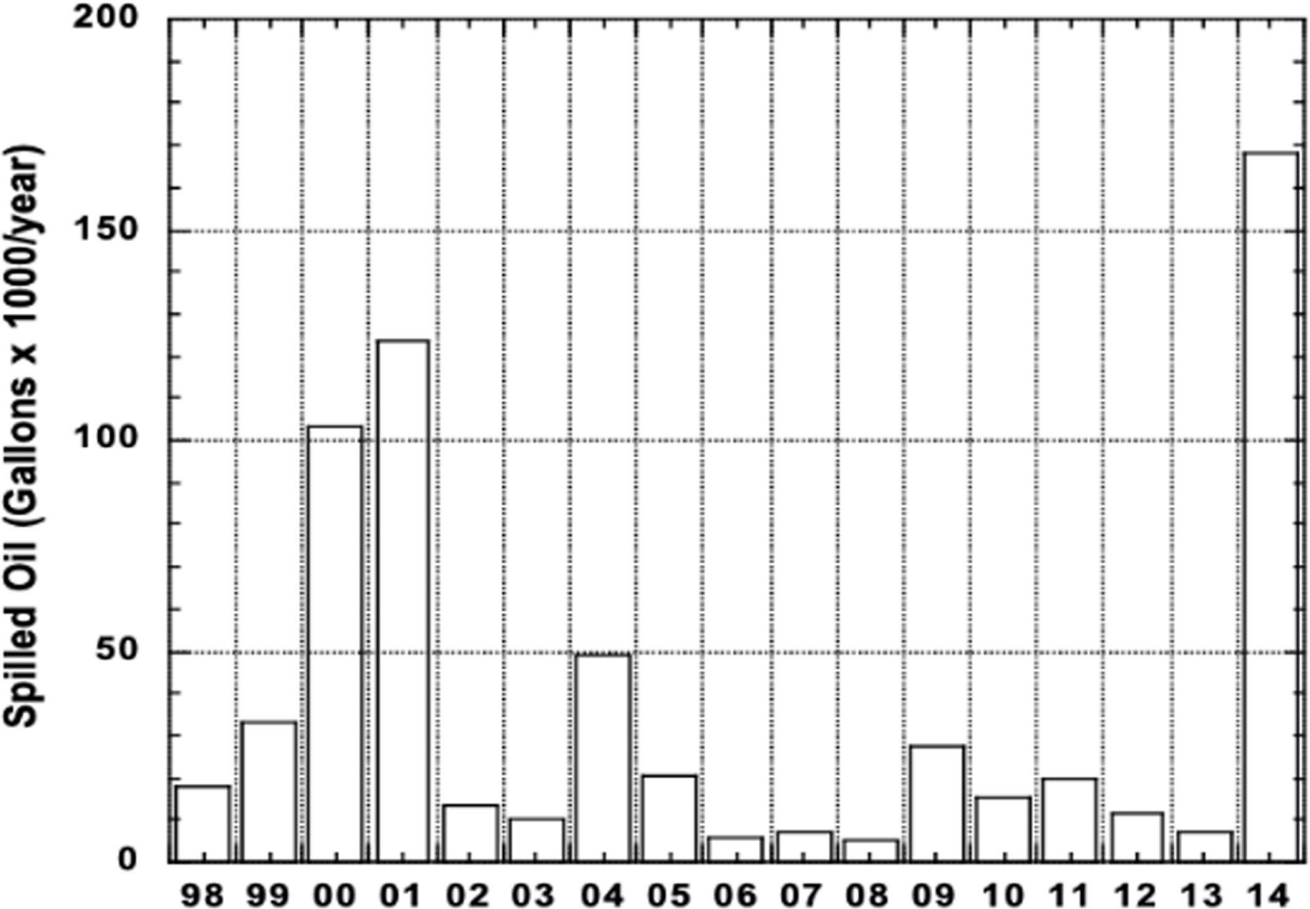
Self-reported oil and gas spills from 1998 to 2014 in Galveston Bay (https://www.harcresearch.org/).

Galveston Bay (GB, Fig 1) covers an area of ca. 600 sq. mi (1544 sq. km) and has an average depth of ca. 2.1 m. The surrounding wetlands add another ca. 150 sq. km. The exceptions to these shallow depths are the Houston Ship Channel, the Inter-coastal Waterway and various harbors and channels scattered around the bay's periphery. Galveston Bay is surrounded by one of the highest concentrations of oil refinery and chemical industry in the US, amounting to 30–50% of the countries capacity. On its north and west margins it is contiguous with the rapidly growing Houston area of ca. 4 million people, the 4th largest urban area of the United States [12]. On the bay's east side are farmlands and wildlife sanctuaries. The extensive Trinity River drainage basin that starts in north Texas near the large city of Dallas and the more limited San Jacinto River that skirts Houston both debouch into the head of the bay. Metropolitan Houston is considered the oil, gas and associated chemical industry capital of the USA because the counties on the north and west margins of Galveston Bay are home to dozens of refineries and chemical plants [12]. From 2005–2010, over 45,000 vessels (~8000 annually) travelled across Galveston Bay [13], but shipping via the Houston Ship Channel has increased with the Panama Canal expansion in 2016. While this shipping increases the chances for large volumes of petroleum products to be released into the bay (e.g., Texas City bunker oil Spill in 2014, [7, 14], most of the spills are small (up to 100 gallons, ~400L). More than 4560 spills were reported in the Lower Galveston Bay watershed by the Texas General Land Office during the period 1998–2014 (Fig 2), with a maximum of 397 spills reported in 2001 and a low of 184 spills reported in 2011 [7], most of them small Galveston Bay dominates the commercial and recreational fisheries in landings and economics of Texas. This includes commercial oysters, blue crab and shrimp fisheries, plus commercial and recreational fin fisheries [12].

The contiguous relationship between urban and industrial waste and fisheries has prompted extensive studies of toxic contaminants and harmful organisms (bacteria, algal blooms, fish species) in the water, sediments and biota of Galveston Bay [15]. These include but are not limited to trace organics such as DDTs, PAHs, Dioxin and PCBs [16–18] and potentially toxic trace metals [12]. The focus of this paper is on the sources, concentrations and fates of PAHs. These materials are of particular interest because they are a significant by-product of combustion, industrial wastes, natural oil seepage and oil spills in and around Galveston Bay. While several studies have been conducted that quantify PAHs and their transformations in or around the bay, few have tried to link them all together to estimate possible impact on human health. Our goal therefore was to trace PAHs from their various sources to their fates, including human exposure, and put it into perspective to that of what we know about other pollutants in the Bay, as well as to PAHs in other estuaries. The general approach in this analysis was to determine concentrations of PAHs in defined biological and physical compartments of the Galveston Bay ecosystem and then estimate loading in each compartment. Following this, an attempt was made to determine fluxes between the living stocks and geochemical compartments, including losses to metabolism, burial, etc. In principle, this was an attempt to balance the total input with the probable fates, an approach used previously in other ecosystems (e.g., [19] in Lake Superior). Finally, estimates are made of the exposure of the general public via seafood products.

## Methods

PAHs in oysters, marcrofauna and fish, were collected from locations in Galveston Bay listed in Table 1 [1, 20] program (S1 Table). In addition. samples of the American oyster, *Crassostrea virginica*, were collected in November–January in selected years from 1986 to 1998 as part of the National Oceanic and Atmospheric Administration (NOAA) National Status and Trends (NS&T) Mussel Watch Project. Samples were collected from six sites in Galveston Bay shown on Fig 1, including the Ship Channel (GBSC), Hanna Reef (GBHR), Yacht Club (GBYC), Todd’s Dump (GBTD), Offatts Bayou (GBOB), and Confederate Reef (GBCR) as described in Qian et al., (2001). Triplicate sediment samples were also collected and analyzed at each site. Sediment and benthic macrofauna samples were sampled from seventy-five sites between 29 July and 16 August 1996 in Galveston Bay, and results are reported by [21].

**Table 1:** PAHs (ng/g wet weight) in seafood samples collected from Galveston Bay.

DATE	LOCATION of SAMPLE		SPECIES*	REPLICATES	AVERAGE	Std. Dev.
1/12/12	Seafood market		Brown Shrimp	3	3.51	3.12
“	“		Oyster	3	4.25	0.96
“	“		Spotted Trout	3	5.06	1.15
“	“		Blue crab	3	1.81	0.70
7/12/12	“		Brown Shrimp	5	38.14	5.19
7/12/12	“		Oyster	5	22.61	7.20
3/23/13	29.2 N.Lat.	94.98 N.Long.	Spotted Trout	5	28.62	9.72
9/28/12	29.16 N.Lat.	94.54 N.Long.	Oyster	5	35.40	3.51
7/8/13	29.48 N.Lat.	94.73 N.Long.	Oyster	6	25.38	6.82
11/10/12	29.18 N.Lat.	94.46 N.Long.	White Shrimp	5	43.05	12.55

*Brown shrimp (*Farfantepenaeus aztecus*), Oyster (*Crassostrea virginica*), Spotted Trout (*Cynosion nebulosus*), Blue crab (*Callinectes sapidus*), White shrimp (*Litopenaeus setiferus*)

In addition to the historical oyster samples, species of seafood analyzed from Galveston Bay (Table 1) were the Spotted Sea Trout (*Cynoscion nebulosus*, 8 individuals), the common oyster (*Crassostrea virginia*, 19 individuals), the brown shrimp (*Farfantapenaeus aztecus*, 8 individuals), and the blue crab (*Callinectes sapidus*, 3 individuals). As indicated (Table 1), some species were purchased at ‘The Seafood Market’ on 61^st^ St. in Galveston, Texas. The white shrimp (*Litopenaeus setiferus*, 5 individuals), Trout (5 individuals), and oysters (11 individuals) were sampled in the Galveston ship channel adjacent to the University of Texas Medical Branch facilities within the city of Galveston. These samples were processed using the QuEChERS extraction method [22] with minor modification as described by Agilent (p/n 5982–6555, p/n 5982–5158). The processed samples were analyzed by GC-MS and using Selected Ion Monitoring mode. Deuterated phenanthrene was used as the recovery standard while deuterated naphthalene, acenaphthene and perylene were used as running standards. The retention times of the PAH’s and their alkylated analogs were obtained using the standard 16 EPA priority pollutant PAH’s and an oil sample, while the conformation of the PAH’s was done based upon retention time [20]. Deuterated phenanthrene was used as a recovery standard. The experiments were performed according to [20], which provides details about detection limits, and recoveries etc. The mean (total) PAHs concentration of the store-bought species was 12.6 ng/gram whereas the mean of the harbor-caught species was 33.1 ng/gram (Table 1), which reflects the high variation in the concentrations measured.

The concentrations of PAHs in the literature are multiplied by the 'stock' size (from various sources, as indicated in the Table 3) to provide total loading on a 'per square meter' basis, not just concentration. This loading per food web category is then expanded to the entire bay. The source terms taken from the literature are assumed to be reasonable and well-estimated, and the fates have been determined, and thus this simplified PAHs budget is an 'inverse' model in that we are attempting to connect the source terms with the fates. The final budget considers market samples that are ostensibly consumed by humans exposed to a range of levels of PAHs from fisheries products.

## Results and discussion

Results that are reported were using data collected in the current study and from the literature. Table 1 reports PAHs concentrations in seafood samples, Table 2 contains PAHs in the compartments (water, sediments to oysters, fish) in units of ng PAHs per gram wet weight. Table 3 has the biomass (or organic matter equivalent) in each compartment in units of grams wet organic matter per square meter (equivalent to mt/km^2^) taken from a wide variety of sources, as indicated. Table 4 combines the two concentrations multiplied by the biomass categories. This information is then expanded by multiplying by 1600 km^2^ for the whole bay in a single budget. Table 5 provides details of the total delivery of PAHs to the public via fisheries products. Finally a box model or flow diagram has been constructed for the entire open bay area, not including wetlands and marshes, which described the sources, concentrations and fates of PAHs.

**Table 2:** PAHs in Galveston Bay (ng/g wet weight).

CATEGORIES	MEAN CONCENTRATIONS*	STD. DEV.
Water^1^	Range of 0.8 to 18.3 ug L^-1^	
Sediment^2^	6.1 to 1885 (surface 1 cm)	1263
Zooplankton^4^	ca. 50	102
Benthic Infauna^5^	48.6	
Oysters^6^	203	68
Shrimp^7^	28.2	17.6
Blue Crabs^8^	19.3	13.0
Fin Fishes^8^	4.7 to ca. 24	

*ng/gram wet weight (unless otherwise indicated)

^1^See text for surrogate concentrations (Sinaei and Mashinchian, 2014 [99]), and a breakdown of inputs (Park et al., 2001 [99])); additional data are found in Louchouarn et al., 2018, [29, 45]; Santschi et al., 2009 [23], Bacosa et al., 2020 [17].

^2^Surface concentrations, in μg/g: The State of the Bay Report (NOAA, 2003 [28]; EPA REMAP 2003 [30]; and Santschi et al., 2001 [18], 2009 [21])

^3^See text for discussion of possible ranges

^4^Carls et al. (2005) [26], from congeneric copepods in Port Valdez, Alaska

^5^Based on an amphipod crustacean in the Houston Ship Channel (Soliman and Wade, 2008 [27])

^6^Qian et al. (2001) [30]; compare to 41.1 (39.2) in GBNEP (2003) [48] at Morgan's Point and Eagle Point and 21.9(11.2) in S1 Table

^7^ S1 Table ^8^GBNEP (2002) [53]

^8^GBNEP (2003) [51] and S1 Table

**Table 3:** Biomass in Galveston Bay (g wet weight/m^2^).

CATEGORY	BIOMASS*	STD. DEV.	LANDINGS**	STD. DEV.
Phytoplankton^1^	8.4			
Zooplankton^2^	0.83			
Sediment^3^	1,700 (organic detritus)			
Infauna^4^	23.3	12.8		
Shrimp(3 spp.)^5^	0.011	0.004	1.8	0.40
Oysters^6^	2.8		1.7	0.66
Blue Crabs^7^	0.034		0.76	0.23
Fin Fish(12 spp.)^7^	.0098	0.009	0.76	
Sum	31.2		5.1 mt/year	

* g/m^2^ wet weight or mt wet weight/Km^2^ (ca. 50,000 mt/Bay area of 1600 km^2^)

**Total Mt landings per year for entire bay from SOTB Ch. 9, averaged over the years where data available (blue crabs exhibited a decline over time).

^1^Ornolfsdottir et al. (2004) [21]: assumes mean of 8 ug Chl a/liter, 50 to 1 Carbon the Chl a ratio and 15% dry to wet weight;

^2^Minello and Matthews (1981) [100];

^3^Santschi et al. (2001) [21], grams wet detrital organic matter in top one cm, see text;

^4^Broach (2001) [55] and Qu et al. 2015 [56] (see text);

^5^Fontaine and Neal (1971) [27];

^6^Hofstetter (1987) [58], Anderson (1987) [101], Dekshenieks et al. (2000) [102];

^7^Pullen and Trent (1970) [59].

**Table 4:** PAH loading in categories of organisms in the Galveston Bay ecosystem. (Combination of Tables 1 and 2).

CATEGORY	[PAH]*	BIOMASS**	ng m^-2^
Water [Input of ca. 2 Mt/year (see text)]			
Phytoplankton	8.4		
Zooplankton	50	0.83	42.3
Benthic Infauna	48.6	23.3	1132
Oysters	203	2.8	568
Shrimp	28.2	.011	0.31
Blue Crabs	19.3	.034	0.66
Fishes (12 spp.)	4.7	.0098	0.05
			1,743.3

*ppb wet weight or ng g^-1^ wet weight

**g m^-2^ wet weight or t km^-2^ wet weight

**Table 5:** Total delivery of PAH to the public via fisheries products, determined by multiplying measured concentrations times the landings (Galveston Bay National Estuary Program, 1993).

Sea Food Type	Landings (mt/year)	PAH Loading (grams/year)
Oysters	1.7	345
Shrimp	1.8	51
Blue crabs	0.76	15
Fin Fish	0.76	3.6
***Totals***	5.0	415

A principal input of PAHs to Galveston Bay is via the atmosphere [23]. A total of 2 metric tons yr^-1^ (mt) can be divided into gas exchange, wet deposition and dry deposition, although most of it is gas exchange from the atmosphere. PAHs concentrations ranged from 4 to 161 ng m^-3^ in dry deposition, and from 50 to 312 ng L^-1^ in rain. The annual wet deposition flux was estimated to be 130 μg/m^2^-yr, and dry particle deposition flux to be 99 μg/m^2^-yr. The net gas exchange from the air to the surface water was estimated to be 1,211 μg/m^2^-yr, although this was considered preliminary [23].

Self-reported spills of gas and oil in Galveston Bay have been collected since 1998 [24] The mean number of spills reported from 1998 to 2014 was 268 per year, with the mean volume being 37,500 gallons per year, but this ranged from a low of 4,900 gallons up to 168,000 gallons per year (Fig 2). The latter high value was associated with a single spill in March of 2014 at the entrance to the bay known as the Texas City Y spill [7]. A gallon of oil is assumed to have a specific gravity of ca. 0.9 and thus would weigh 3.4 kg. Of this ca. 0.8 to 1.5% is PAHs [25]; thus the mean PAHs introduced into the bay in the reported spills would range from 1.0 mt to 1.9 mt PAHs/year.

**Fig 3:**
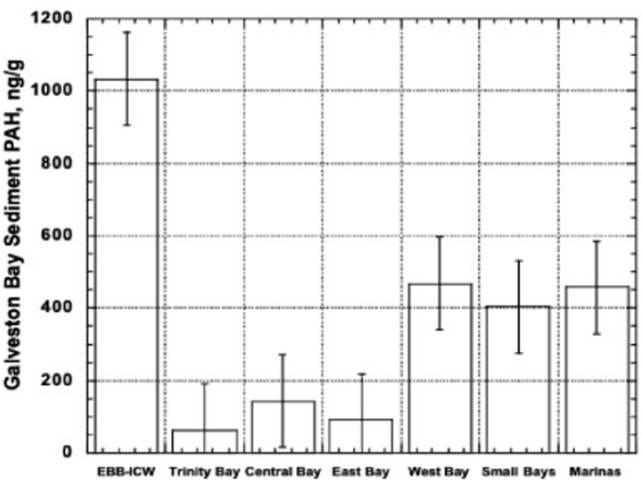
Distribution of PAHs in various habitats of Galveston Bay (EPA REMAP 2003) [60].

The EPA sponsored REMAP survey [26] found a mean value of PAHs in sediments of 364.7 ng/g dry weight, but the values ranged from 6.1 ng/g in the middle of the bay up to 1885 ng/g near an oil well in the center of Trinity Bay. The distribution varied by location (Fig 3). The lowest values were in Trinity Bay but two high values near an oil well have been omitted [26]. The extremely high values were observed in the bayous of the extreme East End of the bay, but in central East Bay the values were modest. In general small bays and marinas all exhibited the highest values of PAHs

**Fig 4:**
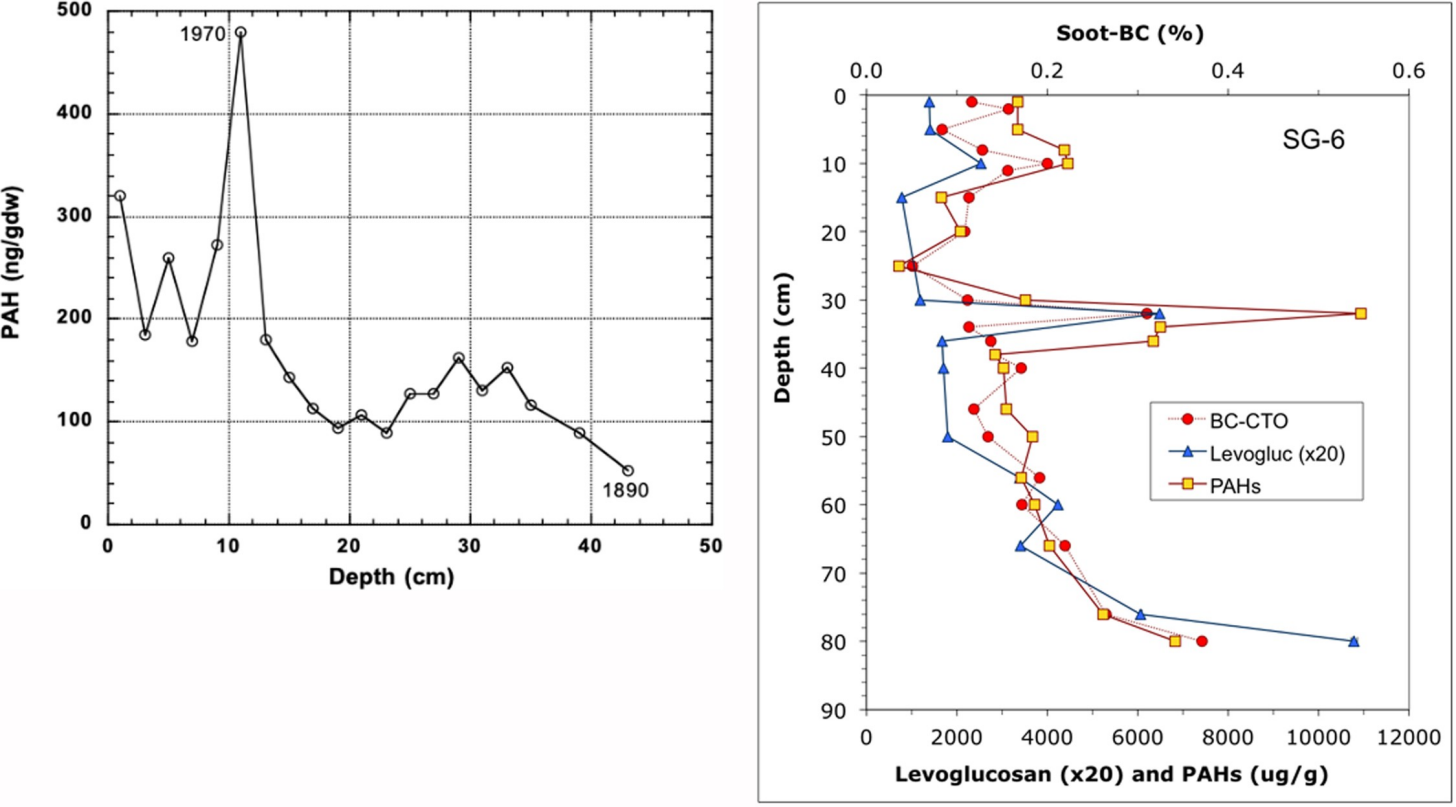
**a.** PAH in Galveston Bay sediment (central Trinity Bay) as a function of depth and time [104]. **b.** Hydrophobic organic contaminants (PAHs), BC geosorbents (Chars and Soot), in Core SG-6. PAH confirm combustion by-products and large hydrocarbon dumping; BC (soot) confirms high-temperature combustion by-products (correlated to PAHs) and levoglucosan confirms biomass low-temperature combustion (correlated to BC and PAH). The trends in co-occurrence of all these markers are unique to this environment and are not seen elsewhere [16]. This figure is from an open access article distributed under the terms of the Creative Commons CC-BY license, which permits unrestricted use, distribution, and reproduction in any medium.

A detailed vertical profile of PAHs within the sediment (Fig 4A) was derived from a core sample taken in the central outer Trinity Bay [27]. Subsamples were taken every few cm's down the core to a depth of ca. 43 cm as shown in Fig 4A. These sediments were also dated, as indicated, using the Plutonium maximum of 1963, providing an accumulation rate in terms of cm per year. The gradient depicts an accumulation from ca. 1890 up to about 1995, with a single exceptionally high value of PAHs at about 1970. Values ranged from ca. 320 ng/g dry weight in 'recent' sediments down to ca. 40 ng/g in the oldest sediment more than a century ago. Similar values of 300–400 ng/g were also obtained in the upper 4 cm of a soil core adjacent to Northern Galveston Bay [28]. The profile from [17, 27] illustrates the increase in accumulation of PAHs over time minus any loss to biogeochemical degradation over that same time period. If it is assumed that little degradation has occurred in the anoxic sediment after burial [29], then most of the gradient reflects the time-dependent buildup over the last century. This core represents a single location in the middle of Trinity bay; this build up was higher toward the north where introduction of PAHs and sediment accumulation would be higher due to inflows from the San Jacinto into the Houston Ship Channel (Fig 4A; [29], SI; [28]), and the Trinity River upstream, compared to lower values to the south with lower sediment accumulation rates and less exposure to PAHs. Fig 4B shows a close relationship of total PAHs with terrestrially derived lignin-phenols, and of Pyrogenic PAHs with soot-Black Carbon (soot-BC), demonstrating both a terrigenous and pyrogenic origin of PAHs from combustion by-products and large hydrocarbon dumping; BC (soot) confirms high-temperature combustion by-products (correlated to PAHs) and levoglucosan confirms biomass low-temperature combustion (correlated to BC and PAH) [16].

[21] reported a mean of 468 ppb /dw (ng/g dry weight) for the entire bay, and thus the value used for burial must be assumed to be an average value for the bay, not a maximum. If the high spike in the 1970's (10 to 12 cm deep) is ignored, a first order build up rate can be calculated over time based on the gradient in Fig 4A [27]. This provides a value for the average accumulation, as indicated.

d[PAHs]/dt = k[PAHs]_initial_

where 'k' is the accumulation rate constant of 0.02/yr using the depth gradient in Fig 4.

The concentration of PAHs per square meter in a layer 1 cm thick can be estimated from the following:

[PAHs]/m^2^-cm = (ng PAHs/g of sediment) x (density of dry sediment/cm^3^) x (1—porosity) x 10^4^ cm^2^/m^2^,

with the assumptions that the porosity (water content) is 0.6 (from Santschi et al. 2001) and the density of the dry sediment is 2.5 g/cm^3^, giving an estimate of 3.2 mg PAHs/m^2^-cm.

Applying the burial rates in [27] of 0.16 g cm^-2^ y^-1^ or 0.29 cm y^-1^, the burial of PAHs would be 1.3 mg PAHs m^-2^ y^-1^ or, for the entire bay, it would be ca. 800 to 1500 kg PAHs y^-1^ (0.8 to 1.5 mt y^-1^). While sediment storage of PAHs is considerable (one order of magnitude) higher in the Houston Ship Channel [17, 30], the area of the Houston Ship Channel is only a few percent of the total area of the bay, and thus, the additional amounts of PAHs would be within the error.

The average accumulation rates of sediment-bound PAHs. in Galveston Bay of 1.3 mg/m-2/yr (3.5 μg/m-2/d) are of the same order of magnitude as those reported by [31] for the northern Gulf of Mexico (2–8 μg/m-^2/d) and Chesapeake Bay [32], but considerably close to the upper end of those reported for the Mediterranean Sea [33–37], as tabulated by [31].

**Fig 5:**
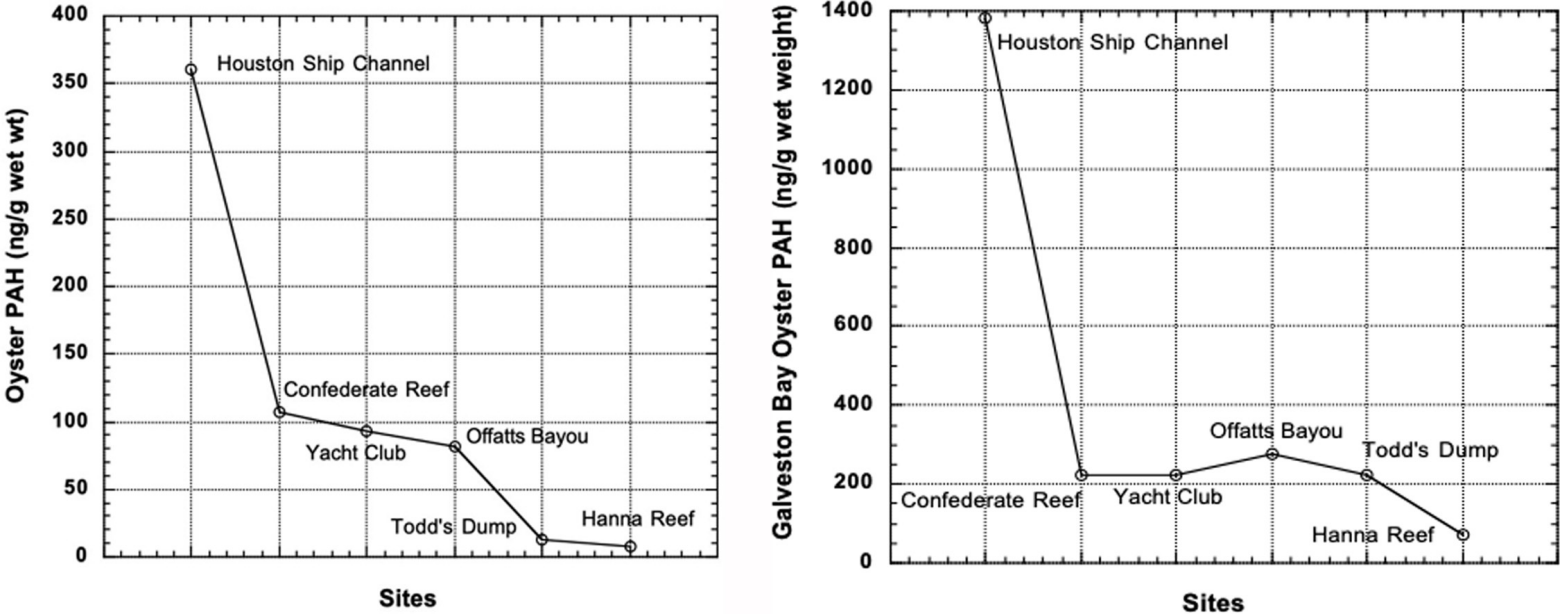
A and B. PAH in oysters in Galveston Bay (A, Galveston Bay Status and Trends data and B, [51]). The ship channel samples were taken at the north end of the bay, whereas the other samples were taken at the south end of the bay near the exit to the Gulf of Mexico (Fig 1). X-Axis is not to scale.

While desorption from the sediment is possible [38], it is not included in this analysis. However, given that the PAHs concentrations in the Trinity Bay sediments reported by [27] are suspended sediments brought by the Trinity River, and the average annual Trinity River sediment load had been reported by [39] as 4x10^6^ mt y^-1^, one could calculate a PAHs sediment deposition rate for Galveston Bay that is similar, i.e., of 1.3 mt y^-1^. There are also inputs from the San Jacinto River and Houston Ship Channel, which left their imprints in the channel sediments [28, 30]. It is doubtful however that much of these PAHs would have made it into Galveston Bay proper, as is evident from the oyster data in Fig 5.

Because phytoplankton are composed of organic matter and have a large surface to volume ratio, considerable PAHs can be bio-concentrated in phytoplankton cells by diffusive sorption [40, 41]. The transfer rate is a function of the concentration of PAHs in the water and the characteristics of the particles, as well as the type of PAHs [42]. Likewise PAHs can attach to particles [43, 44]. The concentration in phytoplankton is likely to be higher than that in the water but is also likely to have a different relative composition of component compounds due to differences in the 'octanol water partition coefficient' [41]. PAHs concentration values in the phytoplankton of Galveston Bay are not presently available. Table 2 contains the range of values presented by [45]. PAHs concentration values in the phytoplankton of Galveston Bay are not presently available.

Zooplankton, like the phytoplankton, can absorb PAHs by diffusion as well, but in addition to bioconcentration they can also bioaccumulate PAHs by eating phytoplankton containing PAHs. Previous studies have not been able to resolve the relative importance of these two processes [11], although there also is indirect evidence that copepods can metabolize PAHs compounds [46, 47]. Lacking values for Galveston Bay, we have utilized values in [48] for *Neocalanus*, a planktonic copepod sampled near Port Valdez, Alaska (mean = 0.051, range of 0.09 to 0.192 ug PAHs g^-1^ wet weight), with a mean wet weight biomass of individual stage 5 copepodites of 2.3 mg per individual.

The concentration of PAHs in phyto-, zooplankton and fish can be parameterized in the following way [49, 50]:

d[PAHs]_p_/dt = {ku _p_ [PAHs] _p_—ke _p_ [PAHs] _p_} for phytoplankton and

d[PAHs] _a_/dt = ku _a_[PAHs] _a_ + {AE x IR x [PAHs] _p_ }—ke_a_ [PAH]_a_ for zooplankton or fish

where ku_p_ is the rate constant for the uptake of PAHs from the water into cells and ke_p_ is the depuration rate constant for the loss of PAHs from the plant cells; likewise ku_a_ and ke_a_. are the rate constants for the uptake and depuration of PAHs into and out of the zooplankton and the fish. AE is feeding rate efficiency of PAHs by either the zooplankton or the fishes; IR is the ingestion rate of PAHs in the prey items.

PAHs bioaccumulate in oysters in Galveston Bay above the concentrations in the surrounding sediments [51, 52]. The original data in the two reports (Fig 5A and 5B) were in nanograms per gram dry weight, but these have been transformed here to wet weight (x 0.15 conversion) in Fig 5 because sea food is sold and eaten wet, both raw and cooked. Note that the [51] values are more than double the [52] concentrations. The general patterns were the same, with the exception of the high value in Offatts Bayou in the [51] (attributable to creosote on pilings). The highest mean values were in the northern apex of the bay in the ship channel; they decreased with distance down the bay toward the gulf. The latter authors noted that in most cases the concentrations were higher in oysters than in the sediments. From their data the bio-amplification averaged ca. 10, but the Ship Channel and Offatts Bayou concentrations are not included (by us) in this average due to their limited area. Bio-amplification of PAHs has been observed in other sediment or particle-feeding invertebrates [51, 53] also noted that oysters preferentially accumulated four to six ring compounds (pyrogenic) rather than smaller two to three ring PAHs (petrogenic, or derived from oil and gas), which were ca. 2.5 times less common in Galveston Bay sediments.

Values for PAHs in shrimp and finfish were measured as part of the [1] S1 Table. Table 1 contains composite samples taken from fish markets at local seafood and bait shop locations adjacent to Galveston Bay from 2012–2013. Those included in this study ranged from 5 to 38 ng/g wet weight for shrimp (S1 Table). No other data on PAHs in shrimp are available for Galveston Bay. Values for the benthic infaunal invertebrates have been extrapolated from a single sample of amphipod crustaceans in the Houston Ship Channel (a sample used to compare other amphipod crustaceans living in the Mississippi Canyon in the deep Gulf of Mexico, [53]. Values for Speckled Trout range from 5–20 ng/g (S1 Table).

**Fig 6:**
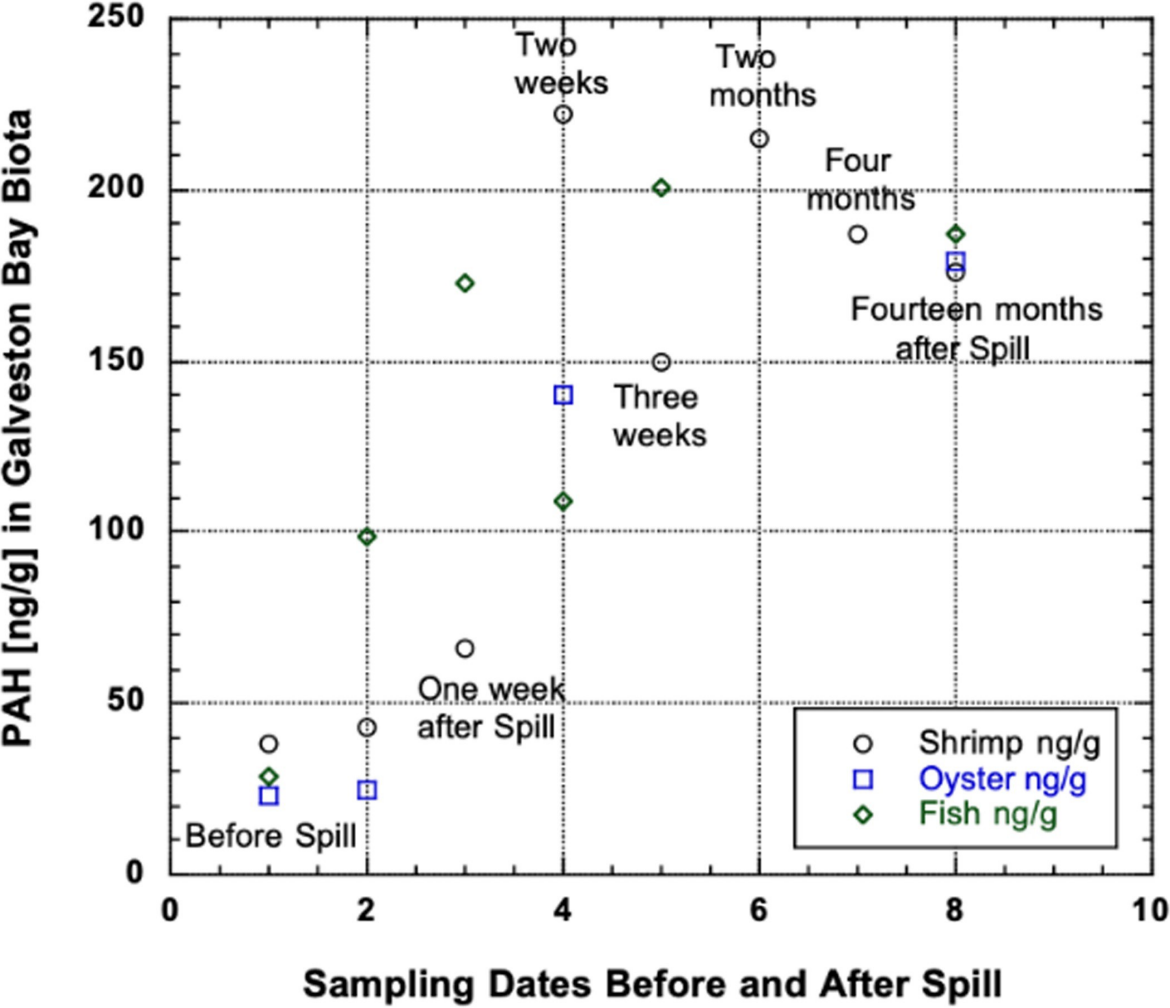
PAH concentrations prior to and after the Texas City ‘Y’ oil spill (2014) near the entrance to the Houston Ship Channel between Galveston Island and the Bolivar Peninsula (from [28]).

The large variation of many values may reflect where in the bay system the specimens were collected (in the channel near UTMB) versus in the bay where the seafood market samples were taken. The large differences could reflect a seasonal variation or the proximity in time and space to a recent spill. Fig 6 illustrates the change in each species over time in relation to a substantial spill. It provides the rate of accumulation over time, followed by depuration after the spill. Furthermore, one would expect to predict bioaccumulation potential from the knowledge of the octanol water partition coefficient, Kow, values. However, experimentally determined relationships between PAH concentrations in fish and Kow values are not straightforward, as previously demonstrated [54]. For example, they determined uptake and depuration rate constants (*k*1) for different PAHs. As they demonstrated, Naphtalene PAHs increased with increasing degree of alkylation and log value of the octanol/water partition coefficient (*K*ow), whereas *k*1 values for three‐ and four‐ring PAHs were lower despite their high log *K*ow values [54].

### Food web biomass and structure in Galveston Bay

The biomass of functional groups in the Galveston Bay ecosystem come from a wide variety of published databases as summarized in Table 3. The phytoplankton standing stock comes from [1]. The usual unit of biomass of phytoplankton is the photosynthetic pigment Chlorophyll a (Chl) and the mean value for Galveston Bay is 8 ug L^-1^ [1]. Given an organic carbon to Chl ratio of 50, the carbon biomass would be 400 ug C L^-1^. If carbon constitutes 40% of dry organic matter, the total dry organic matter in the water would be ca. 1 mg C L^-1^. Assuming the wet biomass is four times the dry weight, then the wet weight biomass of the phytoplankton would be 4 mg wet organic matter per liter or 4 grams of phytoplankton per m^-3^ in wet weight. With an average depth of 2.1 meters, the bay would thus contain 8.4 grams wet phytoplankton per square meter. This is equal to ca. 8.4 mt/km^2^ as well. Thus the total phytoplankton in Galveston Bay would be approximately 13,440 mt wet weight.

The copepod *Acartia tonsa* constitutes on average 50% of the total individuals. The remaining 50% is composed of barnacle nauplii and an assortment of amphipod crustaceans, bivalve larvae, crab zoea, Cnidaria (jellyfish) and Ctenophores (comb jellies) ([1]; Park, 1972 (unpublished data); and [55]). We assume a mean weight of 2.3 mg wet weight per individual, based on [55] for *Pseudocalanus* sp. at a 5th copepodite stage, yielding 0.83 g wet weight m^-2^ (Table 3).

The standing stock of the benthic infaunal invertebrates is based on quantitative grab samples at five locations taken each season over a period of eight years [56]. The locations included Trinity Bay, two down the center of the bay and one in both east and west bay at depths of 1.7 to 3.6 m. Densities averaged 3,335 ind m^-2^ (Std. Dev. = 1831). The polychaete (segmented) worms dominated in numbers (59%) but bivalves (other than oysters) also contributed to the total biomass, though their densities were much lower. An average biomass of 7 mg per ind.- wet weight was assumed, based on the work of Qu et al. (2015) on polychaete worm sizes on the continental shelf. This gives an average value of 23.3 gm wet weight m^-2^ (Std.Dev. = 12.8).

Oyster densities have been derived from [48, 57] with conversion from shell size to wet weights from [58].

### Commercial and recreational landings

The Galveston Bay fisheries landings (Table 5) were assessed primarily using commercial and recreational data collected by the [59, 60]. Seafood dealers are required to provide their commercial harvest of shrimp, oysters, crabs, and marine fish in a reporting system known as the Monthly Marine Products Report. Data were supplemented by trawl and seine data collected by TPWD.

The next step has been to determine the amounts of PAHs in the biota of the bay by multiplying the known biomass values (Table 3) by the known PAHs concentrations (Table 1) in Table 4. PAHs in the biota summed (Table 4) is ca. 1.7 ug PAHs m^-2^ embodied in the biota on average. Thus the 'stored' value would be ca. 2.7 kg of PAHs for the entire bay area. Most of this (> 95%) is contained in the benthic infauna and oysters, with relatively little in motile organisms.

### Oil spills and the dynamics of PAHs in Galveston Bay food webs: Developing a model of sources and sinks

The tabulation of concentrations of PAHs in the standing stocks and landings of Galveston Bay above are represented in the tables as static quantities, The bay is, however, insulted from time to time by medium-sized spills of oil (Fig 2; [61, 62]. The large, most well-documented spills are from collisions between commercial vessels and oil-containing barges (reported in [63]). Opportunistic sampling of the biota after such spills provides information on the 'cycling' of PAHs derived from petrogenic oil in the fisheries, whereas the summary above represents the introduction of pyrogenic PAHs from the atmosphere and the intermittent 'small' spills reported under a broad array of circumstances. Two important sets of large spill data include the *Apex* spill of 700,000 gallons in the Houston Ship Channel at Eagle Point off Redfish Island on July 28, 1990 [7] and the more recent spill of 168,000 gallons of heavy fuel oil at the entrance of the bay from the *Summer Wind* near Bolivar Roads which is referred to as the Texas City ‘Y’ spill of March 2014 (Y [7, 64]). The *Apex* spill study found high PAHs concentrations in liver bile and muscle concentrations one week after the spill but these were back to background levels a month later. As a result of the more recent spill, [65] found PAHs in shrimp, oyster and fish samples (Fig 6) did not return to pre-spill levels fourteen months after the spill.

Pre- and post flooding samples from a industrial neighborhood adjacent to the Houston Ship Channel indicated combustion as the dominant source of considerably higher soil concentrations past flooding due to Hurricane Harvey, and considerable redistribution of soil [14]. Recently, [7] and [14] published PAHs values in the water column across Galveston Bay. The transect run from the mouth of the San Jacinto River to the opening of the bay with the Gulf of Mexico; the values (34–58 ng/L) were on the higher range of those reported in Table 2 from historical data. Immediately after Hurricane Harvey, values in the bay were as high as 180 ng/L which was thought to be associated with storm water runoff on oil and other products [66].

**Fig 7:**
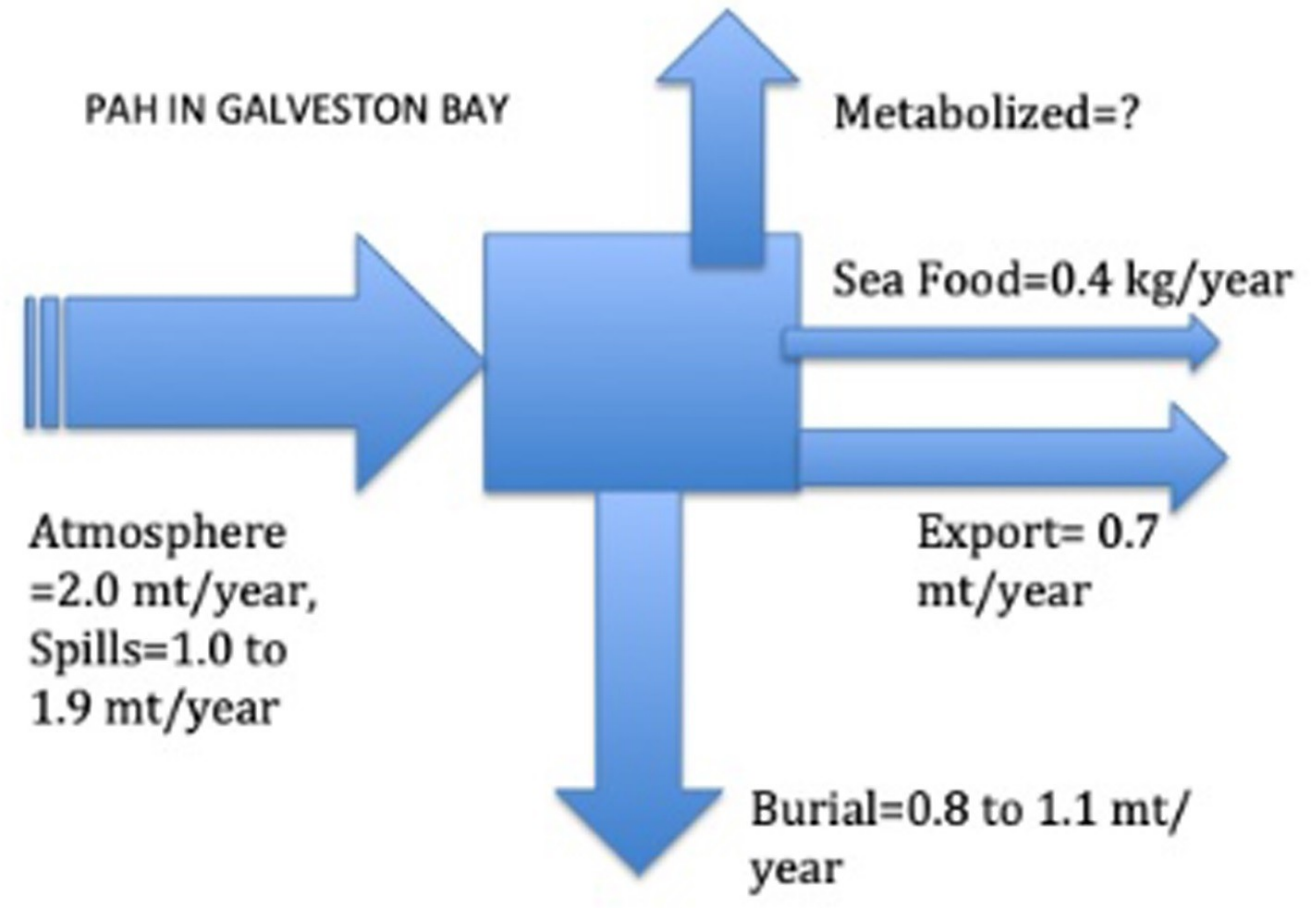
Mass balance of PAH into and out of the entire 1600 km^2^ of Galveston Bay.

A comprehensive mass balance of PAHs was constructed using [18, 64] and the recent measurements (Fig 7). The input to the bay is mostly atmospheric, as indicated [23]. An average of the PAH deposition values determined by [23, 27, 66] were taken and extrapolated over the area of Galveston Bay. The burial appears to be about half of the total loss; it is calculated by extrapolating the single sediment core in [27] to the entire bay. The export assumes that the exchange of water in and out of the bay also carries with it PAHs in the water; as the turnover time of the water is approximately 40 days and the total volume is ca. 3,360 × 10^6^ m^3^, then about 2.5% of the water exits the bay per day. Thus if 2.5% of the input exits the bay each day, we assume that a similar amount of PAHs goes with it. That equates to ca. 0.7 mt/year as indicated (Fig 7). The amount consumed by humans is thus a small fraction of the total (ca. 4 kg/year).

### Perspective to pollutant cycling in Galveston Bay

Galveston Bay is one of the better studied estuaries of the USA, allowing to put the fate of PAHs into perspective of that of other chemicals in the bay. For example, the fate of nutrients [23, 27, 67], potentially toxic trace metals [68–77], trace organics [78–80], particulate and dissolved organic carbon [79, 81, 82], PAHs and other potentially toxic trace organic contaminants [83–85], seasonally varying chl.a concentrations [16, 17], primary productivity [18, 68] and bacterial populations [64, 86], physical mixing [87, 88], as well as sediment delivery, accumulation, and budgets [18, 89] are quite well constrained, with most of these chemicals either cycled and transformed within the Bay, or buried in sediments rather than leaving the Bay. Land-derived terrestrial organic matter inputs and primary and secondary production of exopolymeric substances, combined with intensive wind-induced sediment resuspension in this shallow estuary, lead to an efficient self-cleansing capacity of the water [39, 90] of Galveston Bay. Based on calculated residence times of particles and particle reactive nuclides and substances in the water are of the order of hours to few days [91, 92]. Since most of trace compounds are associated with organic carbon due to its dual capability of binding other chemical species by both hydrophilic and hydrophobic functionalities, and the fact that export of organic carbon and sediment from Galveston Bay is low, e.g., 20 percent or less [93–95], we would have expected that most of the PAHs released to the Bay will be either degraded or buried in the sediments.

## Summary and conclusions

There are several important implications for the information presented. First, the urban and industrial loading of PAHs is split rather evenly between petrogenic and pyrogenic in Galveston Bay; both are large and persistent. That said, ca. 20 to 30% of it is being buried in the sediments but does not reside in the biota. Compared to these totals, a trivially small amount remains in seafood (0.01% or 415 grams, Fig 7). It is, however, surprising that concentrations in biota are not higher than reported, given the highly industrialized area surrounding Galveston Bay, where 30–50% of the USA chemical and oil refinery capacity resides, and the high loading of PAHs to the Houston Ship Channel area. We calculate that about 80% of the PAHs loading still resides in Galveston Bay sediments, but due to their large areal extent, concentrations are moderate. The values for the biota used in the budget were the averages of all the PAH values in Table 1. These were then extrapolated to values for the known population values in the entire bay. Most of the PAHs that is 'stored' in the biota may reside in small organisms that are residents of the sea floor, according to the model, but this estimate has not been validated. This suggests that measuring the loading of PAHs in the smaller biota (infaunal invertebrates and zooplankton) is critical to a better understanding of PAHs cycling. This conclusion is valid for many marine ecosystems adjacent to large urban industrial facilities.

If it is accepted that ca. 20 to 30% of the input of 3 to 4 mt PAHs/yr [96] is being buried in the sediments [97] and a very small portion of the PAHs leaves the bay as fisheries products (Table 5 and Fig 7), then what happens to the 'lost' fraction of PAHs? It may be exported out of the bay by tidal flow (0.7 mt/year, Fig 7) or it may be eliminated by physiological biochemical processes, that is, a suite of metabolic transformations. The latter could be producing alternative PAHs compounds that remain in the ecosystem but which are not analyzed for. There is extensive evidence that many of the vertebrates metabolize PAHs, but the end products are not necessarily known. These unknown products may be more or less toxic than the original PAHs, regardless of origin. Evidence from microcosm experiments using a water accommodated fraction of Macondo oil points to the rapid enzymatic transformation of PAHs to oxygenated PAHs [39], which are often not assessed. Thus, it is highly likely that the missing fraction is metabolically degraded but not necessarily to CO_2_.

The entire stock of buried PAHs throughout the bay could be considered a lurking threat because potentially a very severe storm could resuspend the muddy sea floor down several centimeters. The same could be said for the extensive trawling for bait shrimp within the bay [51]. Both actions could put a significant quantity of pyrogenic PAHs into the water column within a few hours. The impact of this is not known, but as has been shown by [27], resuspended material by trawling is settling down rapidly after disturbance. Furthermore, it is presumed that the degradation rate in an anoxic environment is extremely low [98]. But on resuspension the buried PAHs would be presented with a highly oxygenated environment, once again exposing the PAHs to potentially more rapid degradation.

## Supporting information

S1 Table PAHs in seafood collected from Galveston Bay. (PDF)
