# Supplementary material for: Polycyclic aromatic hydrocarbons (PAHs) cycling and fates in Galveston Bay, Texas, USA
Source: PLoS One. 2020 Dec 28;15(12):e0243734. doi: 10.1371/journal.pone.0243734 (PMC7769252; doi:10.1371/journal.pone.0243734)
Supplement: S1 Table — (PDF) [file pone.0243734.s001.pdf]

Supplemental Table 1 PAHs in seafood collected from Galveston Bay.

12-Jan	Gal	from store		Shrimp	3	3.51	3.12
12-Jan	Gal	from store		Oyster	3	4.25	0.96
12-Jan	Gal	From Any		Trout	3	5.06	1.15
12-Jan	Gal	from store		Crab	3	1.81	0.70
Jul-12	Gal	from store		Shrimp	5	38.14	5.19
Jul-12	Gal	from store		Oyster	5	22.61	7.20
3/23/2013	UTMB	29.20.55	94.97.77	Trout	5	28.62	9.72
9/28/2012	UTMB	29.15.55	94.53.57	Oyster	5	35.40	3.51
7/8/2013	UTMB	29.48	94.73	Oyster	6	25.38	2.54
11/10/2012	UTMB	29.18.470	94.46.256	W. Shrimp	5	43.05	12.55

12/13/2011	UHN	29.28401	90.369093	Oyster	5	16.39	8.33
12/16/2011	UHN	no data		Oyster	5	4.28	3.45
1/12/2012	UHN	29.591046	89.642561	Oyster	5	5.07	3.27
1/14/2012	UHN	29.2215	91.13003	Oyster	5	12.43	6.70
1/11/2012	UHN	Area 13		W. Shrimp	5	4.17	1.81
1/9/2012	UHN	29.466711	90.367522	Trout	2	2.37	0.49
1/9/2012	UHN	29.466357	90.357051	Crab	4	1.51	1.07
1/12/2012	UHN	29.554103	89.619228	Crab	3	1.20	0.24
1/14/2012	UHN	29.21832	91.1326	Crab	4	1.26	0.81
6/15/2012	UHN	12:44:46		B. Shrimp	5	35.55	13.52
6/15/2012	UHN	12:44:46		W. Shrimp	5	19.79	6.08
6/15/2012	UHN	17:13:27		B. shrimp	5	77.57	39.00
6/21/2012	UHN	29:52:758	89:38:242	B. Shrimp	5	107.87	24.33
6/15/2012	UHN	11:38:37		Oyster	5	18.50	17.71
6/21/2012	UHN	29:51:29	89:40:40	Oyster	5	32.16	22.59
6/15/2012	UHN	11:38:37		Crab	5	37.36	22.06
6/22/2012	UHN	29:51:29	89:40:40	Crab	3	41.10	31.70
7/1/2012	UHN	29:18:340	88:736:849	R. Snapper	3	18.97	8.04
7/1/2012	UHN	29:18:340	88:736:849	Grouper	3	9.13	8.35
10/15/2012	UHN	29.24.970	90.26.874	Red Fish	1	19.29	
10/18/2012	UHN	29.24.970	90.26.874	Flounder	1	15.61	
10/26/2012	UHN	cut off		Trout	2	28.25	1.60
10/26/2012	UHN	cut off		Trout	2	19.90	4.95
10/14/2012	UHN	29.22.314	90.28.345	Trout	1	16.26	
10/11/2012	UHN	29.22.092	90.25.031	Oyster	5	56.19	25.50
10/25/2012	UHN	29.43.476	89.36.343	Oyster	5	33.74	3.66
10/20/2012	UHN	29.20.193	90.35.613	Oyster	5	71.34	9.62
11/1/2012	UHN	30.04.794	89.29.806	Oyster	5	56.30	9.19
10/11/2012	UHN	29.20.104	90.22.277	W. Shrimp	5	34.39	4.08
10/18/2012	UHN	29.24.970	90.26.874	W. Shrimp	5	28.53	12.31
12/10/2012	UHN	30.158.333	89.472.917	W. Shrimp	5	62.05	6.71
10/11/2012	UHN	29.21.924	90.23.796	Crab	5	77.17	18.52
10/23/2012	UHN	29.23.127	90.28.985	Crab	5	57.98	12.66
12/10/2012	UHN	30.158333	89.472917	Crab	3	48.40	22.54
5/3/2013	UHN	29:25:940	90.22.389	Crab	3	25.72	11.78
5/3/2013	UHN	29.24.586	90.24.168	Crab	3	24.98	9.83
6/13/2013	UHN	29.30.713	89.45.956	Crab	5	19.92	5.83
6/13/2013	UHN	29.30.037	89.45.344	Trout	1	33.84	
6/30/2013	UHN	30.08.27	89.47.81	Flounder	1	32.41	
6/13/2013	UHN	29.30.037	89.45.344	W.Shrimp	5	26.76	13.53
6/13/2013	UHN	29.30.713	89.45.956	B. Shrimp	5	24.39	3.53
6/30/2013	UHN	30.08.27	89.47.81	W.Shrimp	5	32.10	12.47
6/30/2013	UHN	30.08.27	89.47.81	B. Shrimp	5	29.63	2.72
